# The Effect of COVID-19 on Sleep Quality and Mental Health: Adolescents Are More at Risk Than the Elderly

**DOI:** 10.3390/brainsci12111543

**Published:** 2022-11-14

**Authors:** Luigi De Gennaro, Serena Scarpelli, Maurizio Gorgoni

**Affiliations:** 1 Department of Psychology, Sapienza University of Rome, Via dei Marsi 78, 00185 Rome, Italy; 2 Body and Action Lab, IRCCS Fondazione Santa Lucia, Via Ardeatina 306, 00179 Rome, Italy

After the appearance of a novel coronavirus (2019-nCoV) during 2019, the virus has spread with alarming speed and a pandemic quickly developed. The complex consequences of the pandemic phenomenon, i.e., the consequences of the pandemic per se and the countermeasures adopted to control infections and deaths, were associated with a negative impact on sleep quality [1] and, in general, mental health [2]. With a global prevalence of sleep disturbances of approximately 30–40%, as suggested by meta-analytical studies [3,4], a stable association with psychological distress has been repeatedly reported [5].

In Europe, Italy was the first country to report high rates of infection and deaths, and, as a consequence, the Italian Government declared unprecedented restrictive measures with a total lockdown on 9 March 2020. Not surprisingly, many studies on the effects of the lockdown and/or the pandemic were conducted in Italy (e.g., [6,7,8,9]), reporting an increase in sleep difficulties associated with the pandemic, particularly during the lockdown periods. Along this vein, the Italian study by Amicucci et al. [10] also investigated the impact of the COVID-19 lockdown during Spring 2020 on sleep quality and mental health. The main merit of this study is a specific focus on two at-risk groups: late adolescents (18–20 years) and the elderly (65–75 years).

The authors used a web-based survey and validated questionnaires to assess sleep quality, insomnia, stress, depression, and anxiety. The adolescents reported more insomnia symptoms, worse sleep quality, longer sleep latency, higher daytime dysfunction, a more prevalent disruption of sleep habits (bedtime, get-up time, and nap), and a more negative impact on mental health (higher levels of depression and perceived stress than the elderly). Older participants showed shorter sleep durations, lower habitual sleep efficiency, and greater use of sleep medications. These findings in the elderly are mostly common characteristics of sleep in this age range [11], and they can be considered to have been pre-existing prior to the pandemic. On the other hand, the results from the late adolescents point to pervasive consequences for sleep and mental health in this group.

The apparent paradox is represented by the elderly population having the highest risk of morbidity and mortality due to the pandemic, whereas adolescents show the strongest impact in terms of sleep quality and mental health. This finding should be considered in light of previous observations that point to greater resilience in older adults during difficult times [12], which is likely associated with better emotional regulation and coping strategies [13,14] developed over one’s lifetime. Although this study does not allow for the disentanglement of the consequences of the pandemic per se and the consequences of home confinement (i.e., the COVID-19 lockdown), the interpretation in terms of a greater suffering due to the restrictive measures experienced by adolescents seems more tenable, since about sixty percent of adolescents reported the restraining measures having a negative impact on sleep as compared to about forty percent of the elderly. However, a note of caution with regard to the interpretation of these findings is needed, since several pieces of information about the composition of the sample (e.g., job, family composition, social environment, physical activity, and lockdown- or pandemic-related changes in daily habits) are not available and may have affected the results. Longitudinal observations and a greater focus on the possible intervening variables are needed.

Another point highlighted by the present study appears relevant from a methodological standpoint for future evaluations of subjective sleep quality during the pandemic. Indeed, in spite of a significant difference in insomnia levels between late adolescents and the elderly, the authors did not find differences in the overall subjective sleep quality expressed by the Pittsburg Sleep Quality Index (PSQI) [15] global score. However, this finding is due to a dissociation in the direction of the differences between groups in specific sleep domains that clearly emerged when assessing the PSQI subscales. Consistent with previous observations [16], this result highlights the complex nature of the pandemic’s influence on sleep, and the exclusive assessment of global sleep quality measures may possibly prevent findings from reflecting this multifaced effect.

Overall, the results from Amicucci and coworkers [10] confirm the emerging evidence of late adolescents/young adults having a high vulnerability to pandemic-related sleep and mental health problems (e.g., [8,17,18,19]). In this view, it is crucial to monitor this population over time to detect possible long-term consequences of the pandemic on sleep and psychological wellbeing, determine specific protective and risk factors, and develop timely preventive and interventional strategies.
